# Cataract Operations—Report of One Hundred and Three Cases

**Published:** 1904-10

**Authors:** T. M. McIntosh

**Affiliations:** Thomasville, Ga.


ATLANTA

### Journal-Record of Medicine.

Successor to Atlanta Medical and Surgical Journal, Established 1855, and Southern Medical Record, Established 1870.

VOL. VI. OCTOBER, 1904. No. 7.

BERNARD WOLFF, M.D., EDITOR, Nos. 319-20 Prudential. Published Monthly. M. B. HUTCHINS, M.D., BUSINESS MANAGER, 1007-1008 Century Bldg.

### ORIGINAL COMMUNICATIONS.

### CATARACT OPERATIONS—REPORT OF ONE HUNDRED AND THREE CASES.

BY T. M. McINTOSH, M.D.,
THOMASVILLE, GA.

In these operations there has been no departures from the usual methods of operating, except that the last eight cases, but one, there were made, on the four different persons, operations on both eyes at the same time. This is contrary to the usual teaching, which is that there shall be an interval of ten days or longer between the operations. The writer has never seen any theoretical good reason why both eyes, in mature cataracts, should not be operated on at the same time, and his experience in these eight operations bears out this belief. Other than this, there is no new idea or method in the report of these cases to be of special interest. It is simply those operations that have come in my way in a general surgical practice, and as individual experiences are of value to that individual, especially the errors, the report of these experiences may be of value to other individuals, therefore public record is given to these operations.

The first seventeen of these operations were reported in 1880, in the *Atlanta Medical and Surgical Journal.* The first twenty-eight were done before the anesthetic properties of cocaine were discovered. With the discovery of the use of cocaine, improved technique and more thorough and careful aseptic details, which should be in every case as carefully carried out as if an abdominal section were to be made, there has been markedly better results. The corneal incision has been made in the corneo-scleral junction, with either the Beer's triangular knife, or Graefe's long narrow knife; an iridectomy has been made, the lens capsule lacerated with the cystotome; the lens removed in the usual way.

In all operations where it is not otherwise expressed, Graefe's knife, an iridectomy, laceration of the capsule, has been the smooth progress of the operation. Where departures from this have been made, either by preference, the exigencies of the case, or unruly conditions of the patient, it will be mentioned with each operation. In the earlier operations the Beer's knife was most used, but later on the greater advantages of the Graefe's knife has made me discard entirely the triangular one, though Schweigger of Berlin, when I visited his clinic some years ago, used in the great majority of his operations the triangular knife.

In the earlier of these operations, also, it was customary to confine the patient to bed, on the back, for a week, in a dark room, remove the dressing daily and inspect the eye, but now they are not confined to bed at all, the room is not darkened, and the first dressing is not removed under a week or ten days. The dressing for both eyes, whether both are operated on or not at the same time, is sterilized gauze, absorbent cotton and a number of turns of the figure of eight bandage around the head. In summing up the results, "good" will mean that the patients could read ordinary print easily, or thread a coarse needle, if too illiterate to read. "Fair" will mean that they could recognize persons at a close proximity or attend to the ordinary household duties. Where portions of lens substance have been left behind, forming with the lens capsule a secondary cataract, this will be mentioned with each operation, a condition in which a secondary operation (discission) could give good vision. Failures will be recorded and the reasons therefor with each operation.

1. Maria J., colored, age seventy-one, Jefferson county, Florida. Beer's knife; iris caught on point of knife, knife slightly withdrawn to disengage it, incision completed smoothly; lens presenting in capsule was removed, no iridectomy, slight prolapse of iris on the third day, which left alone, atrophied. Result good.

2. Mrs. J., white, age seventy-five, Thomas county, Georgia. Feeble general health; Beer's knife, iridectomy, free hemorrhage from cut edges of the iris obscuring lens; extraction difficult from movements of the patient; free bleeding into the vitreous humor, no loss of vitreous; panophthalmitis-phthisis bulbi. Evidently from occurrence of hemorrhage from both the iris and vitreous, there was a predisposition to hemorrhage in this case.

3. China H., colored, age sixty, Madison county, Florida. Left eye, Beer's knife, lens presenting was removed, without an iridectomy.

4. Right eye of No. 3. Beer's knife, iridectomy; small amount of lens substance remained, which was absorbed. Result in both eyes good.

5. Mrs. E., age fifty-five, Madison county, Florida. Beer's knife, lens presenting in incision was removed in capsule, slight loss of vitreous; on second day prolapse of iris, which was clipped off and pressure made with bandage. Result good.

6. Captain L., age sixty-five, Madison county, Florida. Beer's knife; an iridectomy; some hemorrhage in the anterior chamber. Result good.

7. Captain T. R. B., age seventy, Clay Hill, Georgia. Pupil sluggish in response to atropine, only partial dilation. Beer's knife; third day some slight iritis: prolapse of iris from one angle of the incision; iritis persisted for about three weeks. Result fair.

8. Thomas B., age seventy, Thomas county, Georgia. Beer's knife; iridectomy; hemorrhage into the anterior chamber. Result good.

9. Elsie J., colored, age fifty-five, Thomas county, Georgia. Beer's knife; iridectomy. Result good.

10. Randall H., colored, age sixty, Thomas county, Georgia. Beer's knife; fluid vitreous escaped immediately upon completion of incision. No iridectomy; lens removed. No benefit.

11. Left eye of No. 10. Beer's knife; fluid vitreous escaped as in the first eye upon completion of corneal incision. No benefit. Both eyes were hydrophthalmic, the result of traumatic cataract of the left eye ten years ago, with resulting sympathetic inflammation in the right. There was perception of light in both eyes and it was hoped there might be some benefit, and the old negro pleaded earnestly for the chance of that hope.

12. Jane, colored, age thirty-five, Thomas county poorhouse. Blind of both eyes with syphilitic irido-choroiditis. Apparently cured of constitutional taint. Left eye, perception of light about one-fourth. Complete posterior synechia, with complete occlusion of pupil by lymph deposits. Operation Graefe's knife; iridectomy piecemeal; lens extracted in the usual way. Cornea flattened, but regained its normal curvature in a few moments. Healing satisfactory, but the pupil closed with lymph. After some weeks Graefe's knife was passed through center of the anterior chamber from side to side, withdrawing knife till point was in pupil site, point was thrust through the occluding membrane into the vitreous, then through each lateral corneal opening, a blunt hook was inserted and hooked into the cut in the membrane and pulled apart, thus making a good central pupil, and the result was good enough for the woman to do housework. The other eye was atrophied.

13. Mrs. W., age sixty, Live Oak, Fla. Prolapse of the iris the second night after the operation; left alone, atrophied of itself. Result good.

14. Mrs. G., age sixty, Thomasville, Ga. Left eye, Beer's knife, iridectomy; bleeding from the iris, removal difficult from too small incision; slight loss of vitreous from pressure required to remove lens; some soft lens material left, which caused a secondary cataract with closure of pupil; there was some corneitis; secondary operation with needles gave fair vision.

15. Left eye of case No. 14. Healing was prompt and result good. This case lost vision after two years from secondary glaucoma, both eyes.

16. Ringold G., age fifteen, Decatur county, Ga. Soft cataract in both eyes. The operation with the needle was required four times. After second operation there was iritis with hypopyon; paracentesis cornea was done. Result good.

17. Mr. C., age sixty, Decatur county, Ga. Right eye, slight loss of partially fluid vitreous before lens was removed, but this was removed with but little more loss of vitreous. Result fair.

18 and 19. Charles S., colored, Dixie, Ga., age twenty-six. Congenital cataract of both eyes. Needle operation, each eye requiring three different operations. The result was so that he could see to walk and recognize people near by, with both eyes. The lack of better vision in this case was due to the long, quiescent function of the retina, which should have been operated on when a child, before the retina lost its function.

20. Mrs. W. B., Madison county, Fla., age fifty-five. General anesthesia with chloroform was demanded by this patient. The operation was completed with perfect satisfaction and lid speculum removed. Before the dressings could be applied patient vomited and from the straining incident thereto there was loss of vitreous. A thin membrane blocked the pupil, through which an opening could be made and thus give good vision. This was declined because it would necessitate a stay of several weeks longer.

21. Luther B., Camilla, Ga., age eight. Soft cataract; needle operation. Some days after operation severe illness in member of the family led the father to take the boy home, a journey of fifteen miles from the depot, of a winter night; resulted in a severe inflammation and total loss of the eye.

22. Rev. E. K. B., Concord, Fla., age sixty. Beer's knife; iridectomy. Patient nervous and unruly; difficult extraction of lens; total loss from suppurative corneitis. Septic infection.

23. Curtis C., Dixie, Ga., age seventy-five. Beer's knife; some loss of vitreous, some hemorrhage into anterior chamber from cut edges of the iris. Result good.

24. Mrs. H., Whigham, Ga., age fifty-eight. Beer's knife; iridectomy; slight membrane occluded the pupil; discission with needles three weeks after extraction of lens; result fair.

25 and 26. Mrs. M. B. J., Thomasville, Ga., age sixty-five. Both eyes were operated on at intervals of three weeks. In the right was a slight prolapse of the iris in one angle of the incision, which left alone, atrophied. The left eye healed without accident. Vision good in both eyes.

27. J. B. G., Cairo, Ga., age fifty-eight. Operation one eye, result good.

28. Rev. Jno. M., Whigham, Ga., age sixty. Right eye, slight prolapse of iris which left alone, atrophied. Result good.

29. Dixon C., Thomasville, Ga., age seventy. Right eye, result good.

30. Mary T., colored, Thomasville, Ga., age sixty. Right eye, result good.

31. Arnold W., colored, Cherry Lake, Fla., age fifty-six. Left eye, result good.

32. Left eye of case No. 29. One year later. Some loss of vitreous. Counted fingers readily immediately after operation. Eight days after the operation died of acute pneumonia of four days duration. Visional result would have been good in this eye also.

33. Wm. S., White Springs, Fla., age sixty. Right eye, result good.

34. Miss R., Brunswick, Ga., age fifty. Right eye, result good.

35. J. J. M., Boston, Ga., age sixty. Left eye. Lens capsule remaining formed a secondary cataract. Central opening made with needle; result good.

36. James B., colored, Ashville, Fla., age about fifty-four. Right eye, result good.

37. Left eye of case No. 36. Operation ten days after first one, result good.

38. Capt. T. T. R., Fort Myers, Fla., age fifty-nine. Right eye; some iritis; secondary cataract. Pupil made in the center with needle. Vision 10/20.

39. Jane H., colored, Grooverville, Ga., age sixty-five. Right eye; loss of some fluid vitreous; result fair.

40. Jno. V., Alamo, Fla., age seventy-three. Right eye, result good.

41. Removing eye of case No. 27, one year later. Result good.

42. Mrs. J. M. G., Whigham, Ga., age fifty-five. Right eye. No iridectomy, as lens presented as soon as the corneal incision was completed; result good.

43. Holland H., colored, Jacksonville, Fla., age seventy. Right eye. The old negro removed the bandage the second day after the operation. Rebandaged and on the seventh day bandage was removed and the eye examined. Incision had healed entirely, and vision good. She left for home the next day without my knowledge.

44. Jane R., colored, Thomasville, Ga., age sixty. Left eye, the vision was gone from iritis and corneitis. Right eye, cataractous with complete posterior adhesions. Operation, lens removed, large iridectomy. Sees well enough to walk about readily.

45. James H., colored, Waycross, Ga., age fifty-five. Left eye, result good.

46. Right eye of case No. 45. Operated one week after the first eye; result good.

47. Wm. M., colored, Quitman, Ga., age sixty. Left eye, result good.

48. Right eye of case No. 47. Operation week after the first, result good.

49. Mr. Robert H., Bartow, Fla., age seventy-one. Right eye, result good.

50. Mrs. Mary P., Thomasville, Ga., age sixty. Left eye, result good.

51. J. L. D., Homeland, Fla., age seventy-seven. Left eye, result good.

52. Rachel S., colored, Dixie, Ga., age about sixty. There was slight increased tension in both eyes, due to slight glaucoma. Right eye, there was some post-operative iritis which clouded the pupil. Result, counts fingers readily.

53. Left eye of case No. 52. Operated on two weeks after the other eye; no iritis, result fair. In both the eyes, after the corneal incision and the escape of the aqueous humor, the cornea became shrunken and regained slowly their normal curvature, as the aqueous humor was resecreted.

54. Greenwood S., Boston, Ga., age sixty-five. Right eye, result good.

55. Left eye of case No. 54. Operation two weeks after the first one ; result good.

56. Mrs. S. J. S., Calvary, Ga., age sixty-five. The cornea shrunk after section ; slowly regained curvature ; result good.

57. Geo. D., colored, Quitman, Ga., age sixty. Right eye, result fair.

58. Left eye of case number 57. Operated one week after the first. Some lens substance remained ; left a slightly clouded pupil, with vision sufficient to count fingers. A slitting of this membrane with the needles would have given good results, but the patient was satisfied with existing condition and preferred to go home.

59. Mrs. J. M. G., Ochlochnee, Ga., age sixty-three. Left eye of case No. 42 ; result good.

60. Aaron S., colored, Quitman, Ga., age seventy-three. Left eye, result good.

61. Joseph K., colored, Hamburg, Fla., age sixty-three. Right eye, result good.

62. Left eye of case No. 61. Operation week after first; result good.

63. Hardy S., colored, Quitman Ga., left eye. Corneal incision too small, as was found when lens did not pass out after it was engaged in the incision. One angle of the corneal incision was enlarged with small blunt-pointed scissors, one blade being passed into the anterior chamber ; during these manipulations the lens was dislocated upward beyond the apex of the incision. The lid speculum was removed, the eyelids closed, and by gentle rubbing downward with the fingers over the closed lid, the lens was returned to its normal position and its removal completed in the usual way, but without again replacing the lid speculum. There was no loss of vitreous. Result, a slight secondary pupilary membrane through which the fingers can be counted readily.

64. Right eye of case No. 63. Operation one week after the first. Patient was a little unruly in this operation, but it was completed satisfactorily so far as the operation itself was concerned, but as the lens was removed, the patient made a strong and persistent effort to close the lid, pressing the speculum on the globe of the eye, which caused a protusion of normal vitreous, separating the edges of the incised cornea. The lid speculum was removed, the partially protruding vitreous clipped off; the lids closed and the eye dressed with some pressure on the globe, without a complete coaptation of the corneal incision. In a week's time the eye was opened and the corneal incision had not yet completely closed; the pressure was reapplied, with the eventual result of good corneal healing. The iris was somewhat drawn upward towards the incision; this, with secondary deposit, closed the pupil. An iridectomy would probably make the vision in this eye fair; this, however, the old negro declined, not wishing to remain away long enough to have this done.

65. T. H. M., Waycross, Ga., age sixty-five. Left eye. After the removal of the lens, cornea collapsed, but slowly regained its normal curve. Result good.

66. Annie, colored, Thomas county poorhouse, age fifty-five. Right eye. Result good.

67. Mr. C. H., Cairo, Ga., age seventy-four. Left eye. After removal of lens cornea collapsed. Third day after the operation walked out in sleep and fell from twelve-foot piazza, bruised parts of his body some, but eye result was good.

68. Rev. A. W. C., Thomasville, Ga., age seventy. Right eye. Incision a little small, which necessitated more than the usual pressure on the cornea to remove the lens, which caused a slight loss of vitreous. Result good.

69. Sarah G., colored, Thomasville, Ga., age sixty five. Right eye. Result good.

70. H. D. B., Bartow, Fla., age fifty-four. Left eye. Result good.

71. Henry M., Pelham, Ga., age three years. Congenital cataract, left eye; needle operation.

72. Right eye of case No. 71. Operation with needle; both eyes at same sitting. Parents took the child home to be under the care of their local physician. Result fair, I presume, as they have never returned for further operation.

73. Jno. A., colored, Madison, Fla. Right eye. Slight anterior adhesion from a penetrating injury ten years before. Cataract removed, iridectomy. Result fair.

74. Left eye of case No. 73. Operation one week after the first one; result good.

75. Anna B., colored, Miccosukee, Fla., age fifty-nine. Right eye. Result good.

76. Left eye of No. 75. Operation one week after first. Result good.

77. Miss K., Monticello, Fla., age about fifty. Left eye. Patient exceedingly nervous, strained violently, closing the lid strongly on the speculum, which pressing on the eyeball forced out considerable fluid vitreous. Vision 10/20.

78. Right eye of case No. 77. Operation one week after the first. Slight loss of fluid vitreous. Vision 15/20.

79. Betty T., colored, Thomasville, Ga., age seventy-two. Right eye. Result good.

80. Left eye of case No. 72. Operation one week after first. Result good.

81. Hasty B., colored, Whigham, Ga., age sixty. Right eye. Incision rather small, enlarged by snip of small blunt scissors in one angle of the corneal incision. Slight loss of vitreous. Result good.

82. Left eye of case No. 81. Result good.

83. J. M. A., Dixie, Ga., age sixty-five. Slight loss of fluid vitreous. Result good.

84. Right eye of case No. 83. Operation two weeks after the first one. Result good.

85. Mrs. R. E. R., Madison, Fla., age sixty-eight. Left eye; slight loss of fluid vitreous. Result good.

86. Rev. A. W. C., Thomasville, Ga. Right eye of case No. 68, operated on four years before. Result good.

87. Mrs. R. E. R. Right eye of case No. 85, operated on one year before. After corneal incision, cornea collapsed, slowly refilled. Result good.

88. Jerry E., colored, Branford, Fla., age seventy-four. Right eye. Result good.

89. Edgar W., colored, Thomas county, Ga., age sixty-five. Right eye. Result good.

90. Elijah S., colored, Valdosta, Ga., age sixty. Left eye. Result good.

91. Lucius R., colored, Thomas county, Ga., age sixty-eight. Right eye. Result good.

92. Mary S., colored, Bainbridge, Ga., age sixty. Right eye. Result good.

93. J. S. P., Greenville, Fla., age sixty. Left eye. Cornea collapsed on completion of incision. Result good.

94. Robert T., colored, Quitman, Ga., age fifty-two. Right eye. Result good.

95. Geo. D., colored, Quitman, Ga., age seventy-two. Left eye had been injured by a blow fifteen years ago and the lens was slightly dislocated; perception of light was good. Operation. As soon as iridectomy was made, lens presented and was removed with its capsule entire; after removal of the lens, and the operation was completed, the lens capsule was lacerated and the contents was fluid. There was slight loss of vitreous.

96. Right eye of case No. 95. Operation done at the same time as on left eye. On completion of corneal section the lens presented in the incision with its capsule entire and was removed by grasp of forceps on its presented edge; no iridectomy. This lens was also partially fluid, its capsule being lacerated after completion of the operation, the nucleus being only half the size of the usual cataractous lens. This was the first time that I had ever removed both lenses at the same time, August 12, 1903. Result in both eyes good.

97 and 98. Stephen C., Crawfordville, Fla., age fifty-four. Left eye, operation smooth. The right eye had an opacity on the upper rim of the cornea from an injury several years ago. Operation smooth; the pupil being below the corneal opacity. Result in both eyes good. Both operations done at the same time.

99 and 100. Ellen C., colored, Sunnyhill, Fla., age fifty-seven.

Both eyes operated on at the same sitting. Result in both eyes good.

101. Right eye of case No. 93, operated on six months before. The cornea in this eye collapsed as did also in the left eye, slowly refilled. Result good.

102 and 103. Mrs. W., Thomas county, Ga., age seventy-nine. Pupils of both eyes were slightly dilated, due to slight attacks of glaucoma. These attacks had manifested themselves two or three times in the past six months, by "seeing spangles of lights and sparks," as she expressed it. The cataract had been mature in both eyes for seven years. In both eyes large iridectomies were made at the time of operation, and in both eyes there was a slight hemorrhage into the anterior chamber from the cut edges of the iris. In both eyes the result was good, both operations being done at the same time.

